# Altered zinc homeostasis in a primary cell culture model of the retinal pigment epithelium

**DOI:** 10.3389/fnut.2023.1124987

**Published:** 2023-04-17

**Authors:** Ana Álvarez-Barrios, Lydia Álvarez, Enol Artime, Montserrat García, Imre Lengyel, Rosario Pereiro, Héctor González-Iglesias

**Affiliations:** ^1^Fundación de Investigación Oftalmológica, Oviedo, Spain; ^2^Department of Physical and Analytical Chemistry, University of Oviedo, Julián Clavería, 8, Oviedo, Spain; ^3^Instituto Oftalmológico Fernández-Vega, Oviedo, Spain; ^4^Wellcome-Wolfson Institute for Experimental Medicine, School of Medicine, Dentistry and Biomedical Science, Queen’s University Belfast, Belfast, Northern Ireland, United Kingdom; ^5^Instituto de Productos Lácteos de Asturias, Consejo Superior de Investigaciones Científicas (IPLA-CSIC), Villaviciosa, Spain

**Keywords:** retinal pigment epithelium, cell culture, sub-RPE deposits, AMD *in vitro* model, zinc dyshomeostasis, physical barrier

## Abstract

The retinal pigment epithelium (RPE) is progressively degenerated during age-related macular degeneration (AMD), one of the leading causes of irreversible blindness, which clinical hallmark is the buildup of sub-RPE extracellular material. Clinical observations indicate that Zn dyshomeostasis can initiate detrimental intracellular events in the RPE. In this study, we used a primary human fetal RPE cell culture model producing sub-RPE deposits accumulation that recapitulates features of early AMD to study Zn homeostasis and metalloproteins changes. RPE cell derived samples were collected at 10, 21 and 59 days in culture and processed for RNA sequencing, elemental mass spectrometry and the abundance and cellular localization of specific proteins. RPE cells developed processes normal to RPE, including intercellular unions formation and expression of RPE proteins. Punctate deposition of apolipoprotein E, marker of sub-RPE material accumulation, was observed from 3 weeks with profusion after 2 months in culture. Zn cytoplasmic concentrations significantly decreased 0.2 times at 59 days, from 0.264 ± 0.119 ng·μg^−1^ at 10 days to 0.062 ± 0.043 ng·μg^−1^ at 59 days (*p* < 0.05). Conversely, increased levels of Cu (1.5-fold in cytoplasm, 5.0-fold in cell nuclei and membranes), Na (3.5-fold in cytoplasm, 14.0-fold in cell nuclei and membranes) and K (6.8-fold in cytoplasm) were detected after 59-days long culture. The Zn-regulating proteins metallothioneins showed significant changes in gene expression over time, with a potent down-regulation at RNA and protein level of the most abundant isoform in primary RPE cells, from 0.141 ± 0.016 ng·mL^−1^ at 10 days to 0.056 ± 0.023 ng·mL^−1^ at 59 days (0.4-fold change, *p* < 0.05). Zn influx and efflux transporters were also deregulated, along with an increase in oxidative stress and alterations in the expression of antioxidant enzymes, including superoxide dismutase, catalase and glutathione peroxidase. The RPE cell model producing early accumulation of extracellular deposits provided evidences on an altered Zn homeostasis, exacerbated by changes in cytosolic Zn-binding proteins and Zn transporters, along with variations in other metals and metalloproteins, suggesting a potential role of altered Zn homeostasis during AMD development.

## 1. Introduction

Ageing encompasses a complex and dynamic array of biological changes that lead to the progressive dysfunction of an organism. Improvement in medicine and lifestyle conditions in the last 200 years has doubled human life expectancy in most developed countries, allowing people to live longer and, thus, increasing the burden of age-related diseases and the demand for research about ageing (1). Age is a risk factor for many ocular diseases of high prevalence, such as cataracts, glaucoma, and age-related macular degeneration (AMD) (2). AMD is the leading cause of irreversible blindness in people over 60 years old, and its prevalence increases with age, reporting a 7-fold increase in people aged 80–85 years old compared with individuals of 45–49 years in Caucasians (3), although the prevalence is significantly lower in other populations (4). AMD is characterized by the degeneration of the deeper layers of the retina and surrounding vasculature in the area of the macula, which causes the loss of central vision and, in advanced stages, irreversible blindness (5). Ageing may contribute to AMD onset by hindering normal functions of the retina, and specifically of its retinal pigment epithelium (RPE), which is primarily affected by this macular disease.

Age-related changes that occur in the retina during AMD development include the buildup of cellular debris between the RPE and the Bruch’s membrane (BrM), as a consequence of the slowdown of the proteolytic systems of the retina (6, 7). This extracellular sub-RPE material produces a thickening and decline in hydraulic conductivity. Drusen, are the clinical hallmarks of AMD, although basal linear and laminar deposits might produce more substantial disruption (8). Although in early stages, sub-RPE deposits do not disturb vision, they may induce chronic inflammation (i.e., para-inflammation) and eventually may disrupt the exchange of nutrients and waste products between the RPE and blood vessels of the choriocapillaris, ultimately participating in the progressive degeneration of the RPE cells and the photoreceptors observed in AMD (9). Sub-RPE deposits contain lipids, minerals and abnormal protein aggregates induced by complex molecular mechanisms, including protein misfolding mediated by metals (10–13).

Metal homeostasis is considered a relevant aspect of retinal physiology that is subjected to changes with age and may be involved in AMD pathogenesis (14). Biologically active metals such as Zn, Cu and Fe have essential roles in cellular physiology. Their excess and deficiency, however, can be harmful to the cell. This suggests that there is a powerful homeostatic system that maintains stable levels. Dyshomeostasis can trigger cytotoxicity, aberrant protein aggregation and consequent loss of function, and contributes to free radical production, inflammation and apoptosis (15–18). The concentration of Zn in the RPE also decreases with age, with the decrease of its cellular homeostatic protein, metallothionein (MT), and the Zn influx transporters ZIP2 and ZIP4 (19). These findings suggest that Zn homeostasis in the retina is affected by ageing and, therefore, altered metallostasis could be a risk factor for AMD development considering that proteins/peptides identified in extracellular sub-RPE deposits undergo pathogenic aggregation promoted by metals (20). *In vitro* models can reproduce the formation of sub-RPE deposits under standard culturing conditions and using membrane inserts imposing a physical barrier against movement of RPE secreted material (21–23). Current protocols for culturing and differentiating RPE cells use culture media containing metal-binding proteins affecting the bioavailability of minerals, including Zn, entailing that normal conditions may be in a zinc-inefficient bioenvironment (24, 25). Therefore, the studying changes during cell maturation and sub-RPE deposition of extracellular material provide an experimental platform to study Zn homeostatic variations, as well as other essential metals. The aim of this work was to study the transcriptome, targeted proteome and metallome of an RPE cell model cultured with conventional medium inducing cell differentiation and secreting proteins extracellularly accumulated to improve our understanding of Zn homeostatic changes.

## 2. Materials and methods

### 2.1. RPE *In vitro* model

Commercial human fetal RPE cells were acquired from ScienCell (Cat. No. 6540) at passage 1 (P1). Cells were propagated and frozen in aliquots at P2. In order to carry the experiments below, aliquots of P2 cells were quickly thawed in a water bath and propagated in a Falcon® 75 cm^2^ Flask (Corning Inc., Cat. No. 353136) coated with 2 μg·cm^−2^ poly L-lysine (Innoprot, Ref. PLL) until 80–90% confluence. Culture media consisted of Epithelial Cell Medium (EpiCM; ScienCell, Cat. No. 4101) supplemented with 100 U·mL^−1^ penicillin and 100 μg·mL^−1^ streptomycin (P/S; ScienCell, Cat. No. 0503), 1% epithelial cell growth supplement (EpiCGS; ScienCell, Cat. No. 4152), and 2% inactivated fetal bovine serum (iFBS; ScienCell, Cat. No. 0010). Shortly before reaching confluence, cells were seeded in 12-well Transwell® plates (Corning Inc., Cat. No. CLS3460) at P3 with a density of 142,900 cells·cm^−2^. Transwell inserts were coated with 2% v/v Geltrex® matrix (Thermo Fisher Scientific; Cat. No. A1413202) diluted in Dulbecco’s Modified Eagle Medium/Nutrient Mixture F-12 Ham (DMEM/F12; Sigma-Aldrich, Cat. No. D8437). Cells were cultured in EpiCM for a week, after which media was replaced with Miller medium (26) to induce cell differentiation. Throughout the experiment, culture media was renewed 2–3 days a week and cells were monitored using a Leica DM II LED (Leica Microsystems) optical microscope and the Millicell ERS-2 Voltohmmeter (Merck Millipore, ref. MERS00002) to measure transepithelial electrical resistance (TEER). Cells from a single donor were cultured up to 2 months and harvested for analysis at different time points.

### 2.2. Immunocytochemistry assays

Cell were collected at 10, 21 and 59 days in culture by fixing in 4% v/v paraformaldehyde for 15 min. Then cells were transferred to PBS buffer and stored at 4°C until immunostaining of ZO-1, CLDN19, BEST1 and APOE proteins was conducted. For immunocytochemistry, fixed cultured cells on polyester microporous membrane were placed on microscope slides, permeabilised using 0.25% v/v Triton X-100 for 5 min and then washed for 5 min with PBS 5 times. Permeabilised cells were blocked for 1.5–2 h in 1% v/v BSA and 10% v/v serum obtained from the primary antibody host (goat in the case of ZO-1, CLDN19 and BEST1; and donkey in the case of APOE) in PBS. Then, cells were incubated at 4°C overnight with the primary antibodies in a solution of PBS containing 0.1% v/v BSA and 10% v/v host serum. The primary antibodies used were: 1:100 v/v anti-ZO1 antibody (Thermo Fisher Sci., ref. 617,300), 1:100 v/v anti-CLDN19 antibody (Novus, ref. H00149461-M02), 1:50 v/v anti-BEST1 antibody (Sigma-Aldrich, ref. MAB5466), and 1:100 v/v anti-APOE (Sigma-Aldrich, ref. AB947). The next day cells were washed 3 times for 10 min with PBS, then incubated with 1:500 v/v secondary antibodies conjugated with AF 488 or AF 594 in a solution of PBS containing 0.1% BSA and 5% primary antibody host serum, for 1 h at room temperature. Finally, samples were mounted in DAKO mounting medium (Agilent Technologies, ref. S302380) or Vectashield® Vibrance® Antifade Mounting Medium (Vector Laboratories, ref. H-1700) for visualization with a widefield fluorescence microscope Leica DMI6000 B (Leica Microsystems) or confocal fluorescence microscope Leica TCS-SP8X (Leica Microsystems). For APOE and BEST1 immunoassays, the Vector TrueVIEW reagent (Vector Laboratories, ref. SP-8400) was used to reduce autofluorescence. Similarly, Sudan Black B was applied for 30 min during the immunoassay of ZO1 to reduce autofluorescence.

### 2.3. RNA isolation and sequencing

The RNA from 10, 21 and 59 days RPE cultured cells (3 biological replicates per time) was extracted using RNeasy Mini Kit (Qiagen). After removing the culture media, lysis buffer was added to the cultures. Lysated cells were transferred to RNase-free Eppendorf tubes, and RNA isolation was performed according to the manufacturer’s protocol. The RNA quality control, library construction and RNA sequencing were performed in BGI Genomics (Beijing Genomics Institute, Shenzhen, China). In brief, the procedure was the following: (i) Measurement of concentration, purity and integrity of RNA using the RNA 6000 pico Kit on the Bioanalyzer 2,100 system (Agilent Technologies, CA, United States); (ii) mRNA enrichment and purification using oligo(dT) coupled to magnetic beads; (iii) cDNA library construction involving mRNA fragmentation, cDNA synthesis (generating first-strand cDNA with random hexamer-primed reverse transcription, followed by a second-strand cDNA synthesis), end-repair, A-tailing and adapter ligation, followed by PCR amplification, circularization and rolling circle amplification for DNA nanoball generation, which harbored more than 300 copies of one molecule; (iv) Sequencing using the platform BGISEQ-500. 100-bp paired-end reads were generated and the sequencing data were filtered using the software SOAPnuke (27), version v1.5.2,^1^ developed by BGI. This software removes the reads containing the adaptor, the reads whose N content is greater than 5% and the low-quality reads (more than 20% bases with Phred threshold score < 10); (v) Alignment, using the software HISTAT2 (28) v2.0.4^2^ to align the clean reads to the reference genome (GCF_000001405.38_GRCh38.p12) and check the quality of data; (vi) Bowtie 2 (v2.2.5)^3^ (29) and RSEM (v1.2.12)^4^ (29) were used to carry out the quantitative analysis through the reads count mapping to gene.

### 2.4. Statistical analysis of RNA-seq data

#### 2.4.1. Differential expression analysis

All the statistical analyses were conducted using the online bioinformatic platform Dr. Tom^5^ provided by BGI. Pairwise comparisons were made between age groups. Each group consisted of 3 biological replicas (see Supplementary Table S1 of Supplementary Material). The inter-group differential expression analysis was conducted using DESeq2 (30),^6^ which considers the variability between biological replicates within the same group. Significant DEGs were identified with Q-values (Adjusted *p*-value) ≤ 0.05.

#### 2.4.2. GO enrichment analysis

After differential expression analysis, we performed enrichment analyses of the biological process GO terms for DEGs between age groups. GO terms with Q-value [p-value corrected by bonferroni (31)] ≤ 0.05 were defined as significantly enriched in DEGs. The figures representing the most significantly enriched biological process terms were also obtained through Dr. Tom software.

### 2.5. Multielemental metallomic analysis by mass spectrometry

Cytoplasmic fraction, containing water-soluble proteins, and cell pellets were processed and collected from each cell culture at different time points (10, 21 and 59 days) for the independent quantification of metal and protein levels. To this end, cultures were washed twice with Ca-and Mg-depleted Dulbecco’s Phosphate-Buffered Saline (DPBS; ScienCell, Cat. No. 0303), cut from the plates together with the polyester microporous membrane and transferred to an Eppendorf tube containing Tris–HCl buffer. Cultures were lysed by ultra-sonication to disrupt cell membranes through three cycles of 30 s at 10 kHz on ice bath. Cell lysates were centrifuged at 16,000 g for 15 min, to separate the cytoplasmic fraction (supernatant) from the pellet (cell nuclei and plasma membrane), and stored at −80°C until analysis. In the case of the pellet fractions, samples were additionally mineralized adding 50 μL of HNO_3_ (68% w/w, TraceMetal™ Grade, Fisher Chemical) assisted by the use of an ultrasonic bath (Fisher Scientific, UK) for 30 min, prior to their analysis by mass spectrometry.

Total Na, Mg, K, Ca, Cu and Zn levels were quantified in both cytoplasmic and pellet fractions from the cell culture samples using two ICP-MS instruments: the Agilent 7900 ICP-MS (Agilent Technologies), equipped with a quadrupole mass analyzer and an octopole collision cell; and the double focusing magnetic sector field ICP-MS Element2 (Thermo Fisher Scientific). To reduce polyatomic interferences, the collision cell of the Agilent 7900 ICP-MS was pressurized with He at a flow rate of 4.5 mL·min^−1^. Instrumental settings were daily optimized for maximum sensitivity and control of oxides formation using a multi-element standard solution (1 ng·mL^−1^ of Ce, Co, Li and Y in 2% w/w HNO_3_ in the case of the Agilent 7900 ICP-MS, and 1 ng·mL^−1^ of Ba, B, Co, Fe, Ga, In, K, Li, Lu, Na, Rh, Sc, Tl, U and Y in 5% w/w HNO_3_ in the case of Element2 ICP-MS). Due to volume limitations, samples were introduced into the ICP-MS using a flow injection analysis (FIA) system comprised of a Rheodyne^TM^ six-port valve fitted with a 5 μL sample loop. Samples were quantified in triplicates and 1% w/w HNO_3_ was used as eluent throughout the FIA-ICP-MS system at a flow rate of 0.32 mL·min^−1^. Quantification was done by external calibration using Ga as internal standard. Specific instrumental parameters are summarized in Supplementary Table S2 (see Supplementary Material).

Total protein concentration was determined using the commercial QuantiPro^TM^ BCA Assay Kit (Sigma Aldrich, Cat No. QPBCA) by external calibration using BSA as standard in a concentration of 0 to 30 μg·mL^−1^. Absorbance was monitored using the spectrophotometer PerkinElmer 2030 Multilabel Reader VICTORTM X5 (Massachusetts, United States). Total protein on the cytoplasmic fraction was used to normalize multielemental determinations, expressing concentrations as ng of element per μg of total protein. As for the pellet fractions, standardization was carried out considering pellet weight, expressing concentrations as ng element per μg of pellet sample.

### 2.6. Quantification of H_2_O_2_ levels in culture media

Hydrogen peroxide (H_2_O_2_) levels were measured in the apical and basal culture media of RPE cell cultures at 10, 21, and 59 days, 3 days after renewal of culture media, using the ROS-Glo™ H_2_O_2_ Assay purchased from Promega (Cat. No. G8820). H_2_O_2_ diffused from the cells to the culture media was quantified by the luminescent signals produced by the luciferin/luciferase reaction. The ratio of luminescence in the samples to a blank (culture media of a transwell insert without cells) was calculated, representing the increase in % of H_2_O_2_ in the culture media and standardized to total protein.

### 2.7. Quantification of MT2A by ELISA

The MT isoform 2A was quantified in the cytoplasmic fractions of cell cultures at 10, 21 and 59 days, using a commercial ELISA kit from Cloud-Clone Corp. (Cat. No. SEB868Hu), based in a competitive immunoassay following manufacturer protocol, and diluting samples with Tris–HCl buffer to a volume of 100 μL (maximum dilution was 1:1). Absorbance was monitored using the spectrophotometer PerkinElmer 2030 Multilabel Reader VICTORTM X5 (Massachusetts, United States).

### 2.8. Statistical analysis

Statistical differences in the levels of metals, MT2A protein and H_2_O_2_ among time points were evaluated conducting two-way ANOVA test using Prism 9 software (GraphPad).

## 3. Results

### 3.1. Characterization of human primary RPE cell culture producing subcellular deposits

#### 3.1.1. Pigmentation and morphology of RPE cells

**Figure 1:**
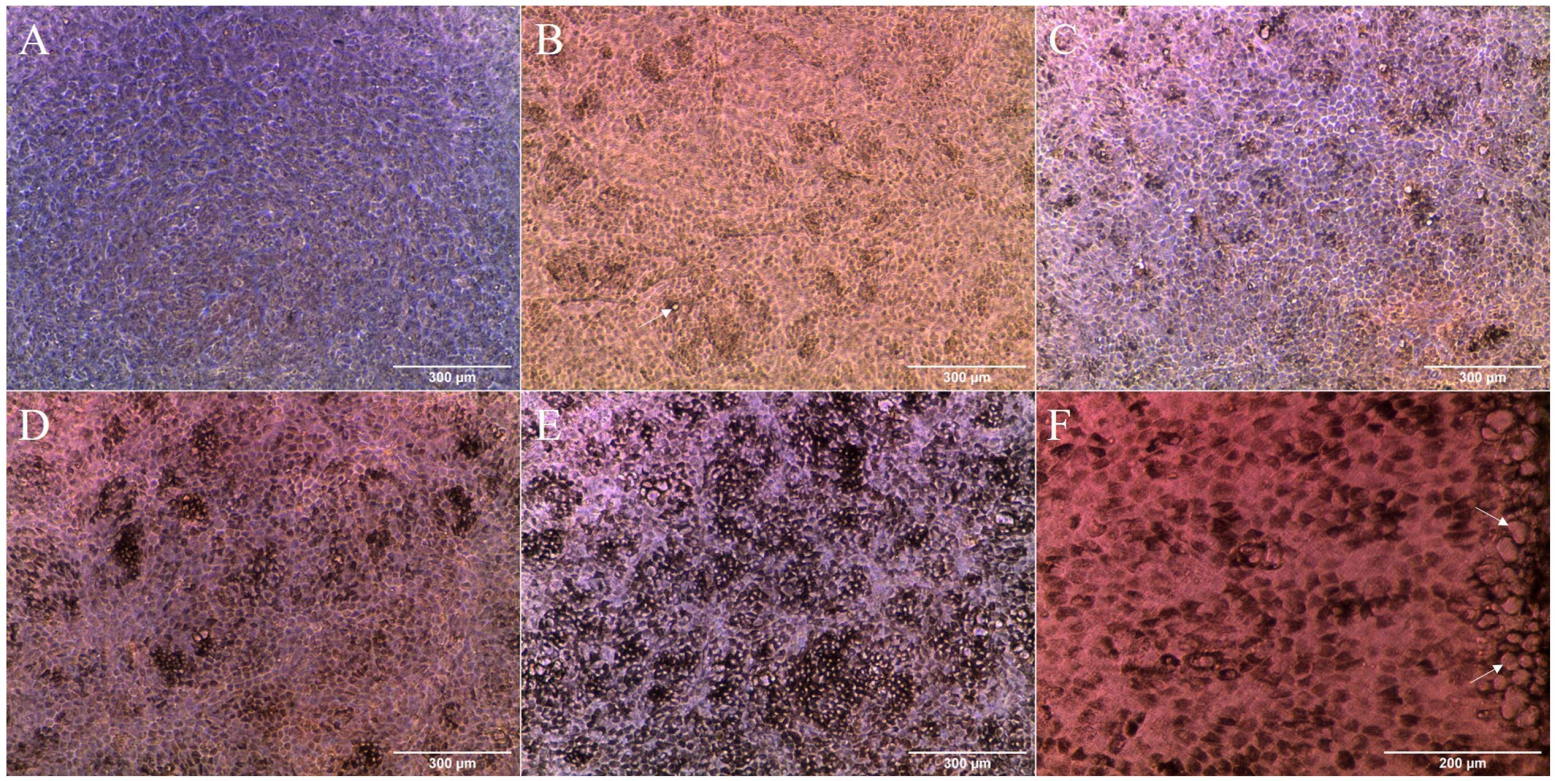
Optical microscopy images of the cell cultures at different time points: **(A)** 3 days; **(B)** 14 days; **(C)** 20 days; **(D)** 28 days; and **(E)** 38 days. Pigmentation of cells started at around 14 days in culture **(B)** and increased with time **(C–E)**. Possible extracellular deposits (arrows) could be seen starting at 14 days in culture (**B**, white arrow). After 38 days in culture, extensive pigmentation could be found throughout the cell monolayer **(E)**. High concentration of possible extracellular deposits (arrows) was detected in the insert borders at 34 days **(F)**.

Morphology and pigmentation were followed by optical microscopy during the maturation of the cell cultures (Figures 1A–E). Slight pigmentation of cells was detected at 14 days in culture (Figure 1B). Afterwards, pigmentation increased from small islets of pigmented cells (Figure 1C) to almost the entire monolayer by the beginning of the second month in culture (Figure 1E). Pigmentation allowed to visualize the cobblestone morphology of cells. Around 14 days in culture, extracellular deposits like structures were visible in the cell culture (Figure 1B), similar to those of mature RPE *in vivo* (21, 32) and increasing in abundance with time. These deposits were mainly concentrated at the edge of the inserts (Figure 1F), where they appeared in groups.

#### 3.1.2. Barrier function

**Figure 2:**
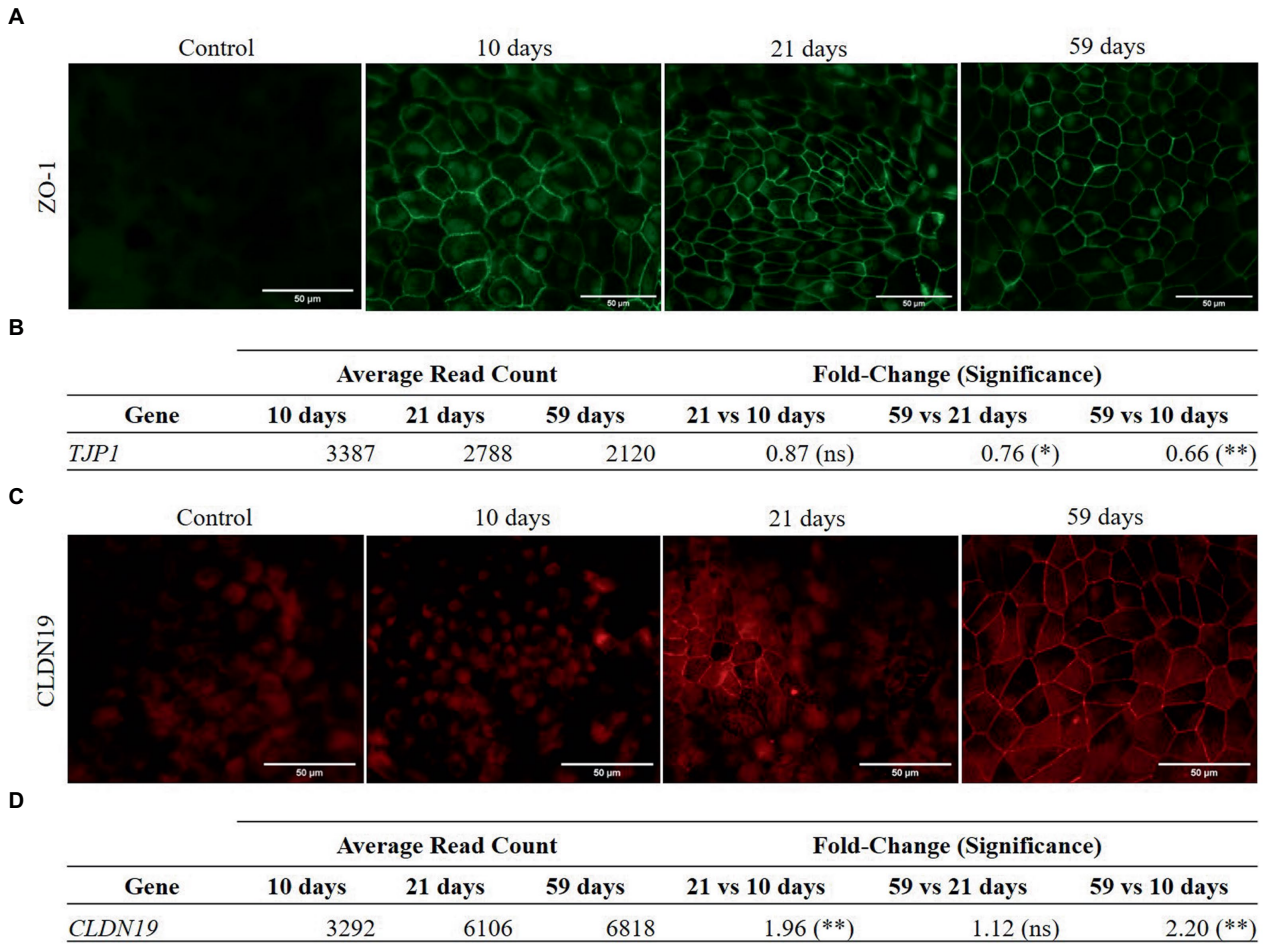
Protein immunolocalization and RNA expression of ZO-1 **(A–B)** and CLDN19 **(C–D)** in RPE cells. Differential gene expression analysis was carried out following the DESeq2 method (ns: *q*-value >0.05; ^*^: q-value <0.05; ^**^: q-value <0.01). **(A)** ZO-1 is detected in the cell membranes of RPE cells at 10, 21 and 59 days in culture. **(B)** ZO-1 coding gene, TJP1, is significantly down-regulated at 59 days in culture compared to 21 and 10 days in culture. **(C)** CLDN19 is not detected at 10 days in culture but appears with increasing abundance in the cell membranes of RPE cells after 21 and 59 days. **(D)** Gene expression of CLDN19 significantly increased from 10 to 21 days in culture and from 10 to 59 days in culture.

Cellular differentiation was monitored through TEER measurement. The barrier function evolution was normalized to TEER values measured at day 3 (see Supplementary Figure S1 of Supplementary Material). Maximal TEER values reached after 1 month in culture (160 ± 60 Ω·cm^2^, range 125–382 Ω·cm^2^). Afterwards, TEER values remained on a pseudo-plateau, with a slight decrease (no significant differences) up to 2 months in culture. Differences in maximal TEER values coincided with the distinct pigmentation pattern of cell cultures. Claudin-19 (CLDN19) and zonula occludens-1 (ZO-1) proteins, involved in forming intercellular tight junctions, have been monitored at protein and RNA expression levels throughout cultured RPE cells (see Figure 2). ZO-1 protein appears early in the maturation of RPE, as it was detected in the membrane of RPE cells at 10 days in culture (Figure 2A). Immunocytochemistry images showed high background fluorescence caused by the presence of autofluorescent granules in the cells (33). From 10 to 59 days in culture, no substantial changes in ZO-1 localization were observed. Orthogonal viewing of confocal microscopy images of 59 days-old RPE cells showed that ZO-1 appeared preferentially in the apical side of the cell membrane (Supplementary Figure S2 of Supplementary Material). Expression of ZO-1 coding gene, TJP1, was higher at 10 days in culture than in later intervals (21 and 59 days) (Figure 2B). In contrast, CLDN19 protein later appeared in the cell cultures, distinguishing signals in the plasmatic membranes of cells after 21 days (Figure 2C). Accordingly, the gene expression of CLDN19 at 21 days was higher than its expression at 10 days (Figure 2D) and did not change significantly afterwards. At 59 days, CLDN19 was detected in most cells by confocal microscopy, specifically in the apical and lateral region of their membranes (data not shown).

#### 3.1.3. Expression of specific markers of RPE

The follow-up of proteins specifically synthesized in the RPE provides an insight into the progress of *in vitro* differentiation of cells in culture. After 59 days, expression levels of important markers of RPE (34–39) increased in the cell cultures (Supplementary Figure S3A of Supplementary Material). Significantly overexpressed genes included markers of the visual cycle (RPE-retinal G-coupled receptor, RGR; lecithin retinol acetyltransferase, LRAT; bestrophin 1, BEST1; retinol dehydrogenase 5, RDH5; retinaldehyde binding protein 1, RLBP1; and retinal pigment epithelium-specific 65 kDa protein, RPE65), genes involved in the biosynthesis of melanin (tyrosine, TYR; dopachrome tautomerase, DCT; tyrosinase-related protein 1, TYRP1; and melanocyte inducing transcription factor, MITF) and the coding gene of SERPINF1 (pigment epithelium-derived factor, PEDF). Immunostaining of BEST1, transmembrane protein strongly expressed in the native RPE, revealed that the protein appears after 21 days of culture in the cell membrane of some cells and becomes more abundant after 59 days (Supplementary Figure S3B). Additionally, BEST1 showed a polarized localization after 59 days in culture observed by confocal microscopy (data not shown), appearing preferentially in the basal area of the cell membrane.

#### 3.1.4. Gene ontology (GO) enrichment analysis

**Figure 3:**
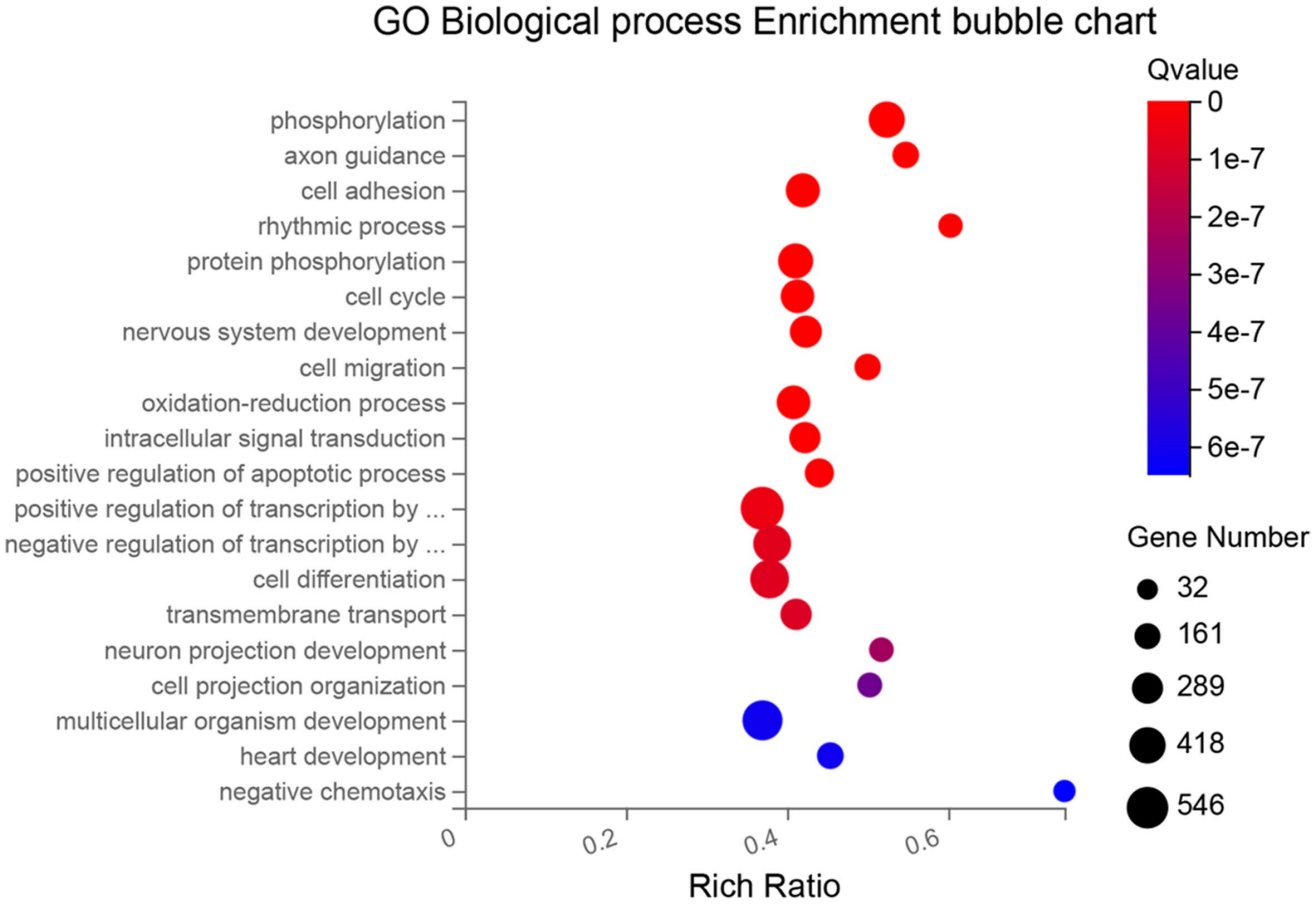
Bubble chart of the 20 most significantly enriched biological processes (GO database) between 10 and 59 days in culture. Each process (in the y-axis) is depicted by a bubble, its size represents the number of DEGs annotated to a GO term, and its color shows the enriched significance (Q-value). X-axis represents the enrichment ratio of each process (ratio of the number of enriched genes annotated to the GO term in relation to the total number of genes annotated to that GO term in the species).

Differentially expressed genes (DEGs) among time points of cultured RPE cells were identified and aligned to the Gene Ontology (GO) database for biological processes enrichment analysis. The top 20 biological processes most significantly enriched in DEGs (including up-and down-regulated DEGs) were selected and depicted as bubble charts in Figure 3. The biological pathways that become altered between 10 and 59 days in culture can be associated with: (1) establishment and maintenance of the RPE (GO terms: “cell cycle,” “cell migration,” “cell projection organization,” “cell adhesion” and “cell differentiation”); (2) the modification of biomolecules, especially proteins (GO terms: “phosphorylation,” “protein phosphorylation” and “oxidation–reduction process”); (3) basic functions of the mature RPE (GO terms: “transmembrane transport,” “intracellular signal transduction” and “rhythmic process”); (4) communication with other structures of the retina (GO terms: “axon guidance,” “nervous system development,” and “neuron projection development”); and (5) temporal progression (GO term: “multicellular organism development”).

#### 3.1.5. Progressive accumulation of sub-RPE deposits

**Figure 4:**
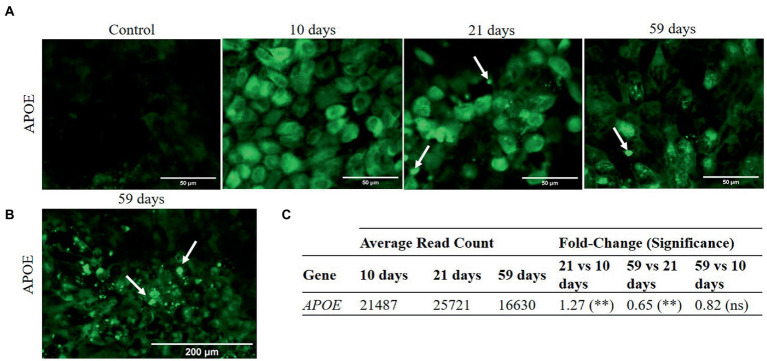
Immunocytochemistry images and gene expression of APOE in cell cultures at 10, 21 and 59 days. Differential gene expression analysis was carried out following the DESeq2 method (ns: *q*-value >0.05; *: *q*-value <0.05; **: *q*-value <0.01). **(A)** APOE is detected in the cytoplasm of RPE cells at 10, 21, and 59 days in culture. Extracellular signals (arrows), possibly linked to deposit formation, were detected after 21 and 59 days in culture (scale bar 50 μm). **(B)** Profusion of extracellular APOE punctate deposition at 59 days (scale bar 200 μm). **(C)** APOE expression increases from 10 to 21 days in culture and decreases afterwards, from 21 to 59 days in culture.

Basal secretion and sub-RPE accumulation of materials have been monitored attending to apolipoprotein E (APOE), other drusen-associated proteins (40, 41), and calcium. The RPE-secreted protein APOE, one of the main components found in drusen facilitating lipid accumulation (21), has been followed up at gene expression and protein localization level along the culture periods. Cytoplasmic signals attributed to APOE protein were found in RPE cultures from 10 days onwards (Figure 4A). Additionally, after 21 and 59 days in culture, we found intense signals in the extracellular space that could be related to extracellular deposits (Figure 4B). RNA analysis showed that the highest APOE gene expression was reached at 21 days in culture and was followed by a significant decrease after 59 days (Figure 4C), although no significant differences were detected when comparing 10 and 59 days, indicative of having reached the initial value.

Genes encoding drusen and Ca metabolism associated proteins (40, 41) were also analyzed at RNA level. Significantly overexpressed genes at the end of 59 days in culture (comparing to 10 days) include the osteoglycin (OGN; fold-change = 11.14, *q*-value <0.001), frizzled-related protein (FRZB; fold-change = 6.23, *q*-value <0.001) and thrombospondin 4 (THBS4; fold-change = 5.76, *q*-value <0.001) (41), which together with the downregulation of biglycan from 21 to 59 days (BGN; fold-change = 0.44, *q*-value <0.001) (42), could be related to the deposition of HAP spherules in drusen, although further evidence is much required. In addition, quantifying Ca levels in the cytoplasmic and pellet fractions of cell cultures could give an insight into the availability of an insoluble form of calcium phosphate, i.e., HAP, and potential extracellular deposit formation. Supplementary Figure S4 compiles the mean values and standard deviation of Ca concentration in both the cytoplasmic (standardized by total protein) and pellet (standardized by weight) fractions of cell cultures. The Ca concentration varied from 1.09 ± 0.24 ng·μg^−1^ (10 days) to 4.01 ± 3.02 ng·μg^−1^ (21 days) and 3.99 ± 0.93 ng·μg^−1^ (59 days) in the cytoplasm, and from 1,399 ± 1,196 ng·g^−1^ (10 days) to 388 ± 50 ng·g^−1^ (21 days) and 1,548 ± 31 ng·g^−1^ (59 days) in the cell pellet, all with no significant differences.

### 3.2. Zinc homeostatic changes during sub-RPE deposits formation

Zn homeostasis was studied over time through its quantitative analysis in cell cultures (both cytoplasm and cell pellet), by ICP-MS, and the analysis of its regulatory proteins, mainly the zinc-binding proteins metallothioneins (MTs) and the zinc transporters.

#### 3.2.1. Total zinc levels

**Figure 5:**
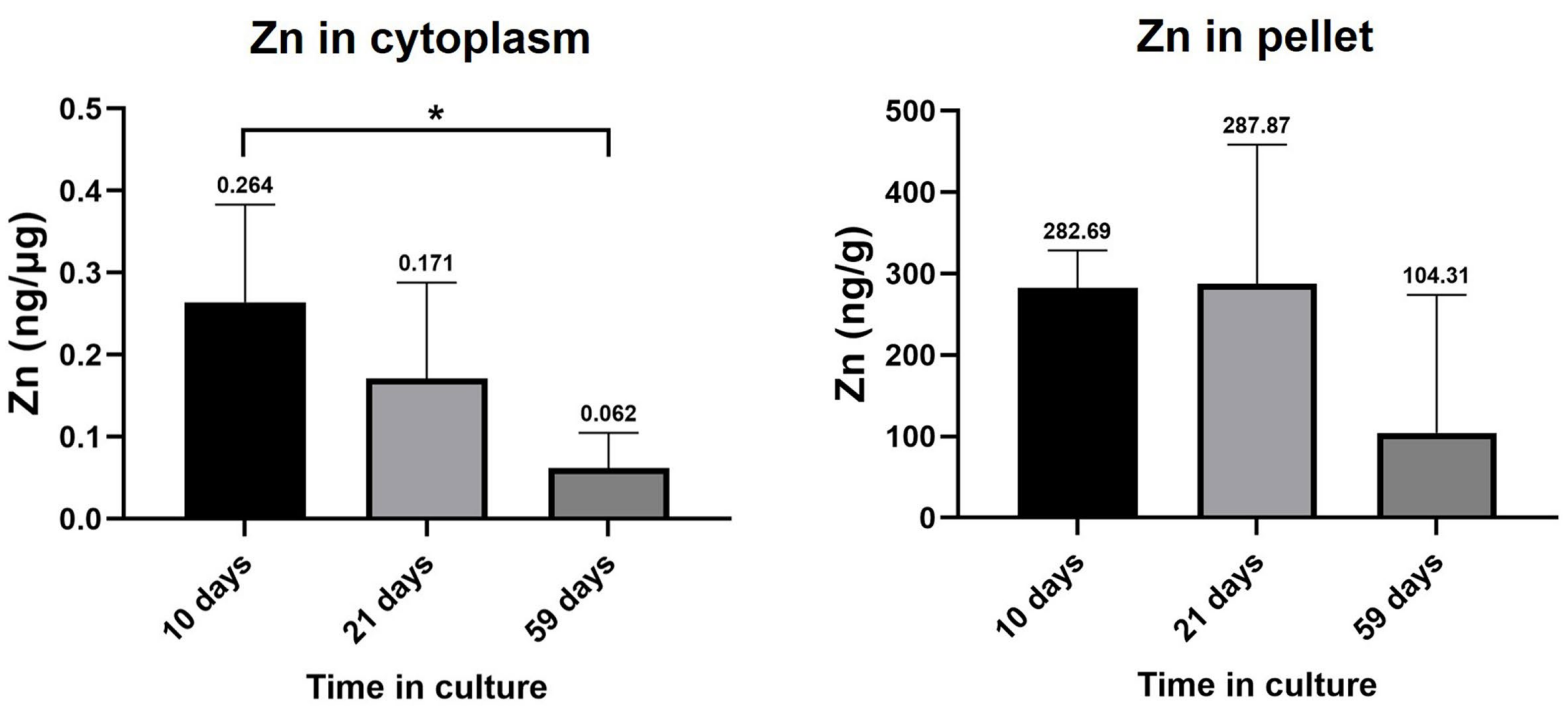
Zinc levels in the cytoplasm and pellet fraction of RPE cells at 10, 21, and 59 days in culture. Data is represented as concentration (ng Zn·μg^−1^ total protein in the cytoplasm; and ng Zn·g^−1^ sample in the pellet) and error bars depict the standard deviation. ^*^: significant *p*-value of two-way ANOVA (< 0.05).

Zn levels in the cytoplasm and pellet of RPE cells at 10, 21 and 59 days in culture are depicted in Figure 5. Cytoplasmic Zn ranged from 0.264 ± 0.119 ng·μg^−1^ (10 days) to 0.171 ± 0.117 ng·μg^−1^ (21 days) and 0.062 ± 0.043 ng·μg^−1^ (59 days), finding a statistically significant decrease between 10 and 59 days in culture (0.2-fold, value of *p* = 0.0293). Zn levels in the pellet fraction ranged from 282.69 ± 45.84 ng·g^−1^ (10 days) to 287.87 ± 170.68 ng·g^−1^ (21 days) and 104.31 ± 169.44 ng·g^−1^ (59 days), without significant changes with time.

#### 3.2.2. Gene and protein expression of metallothioneins

**Figure 6:**
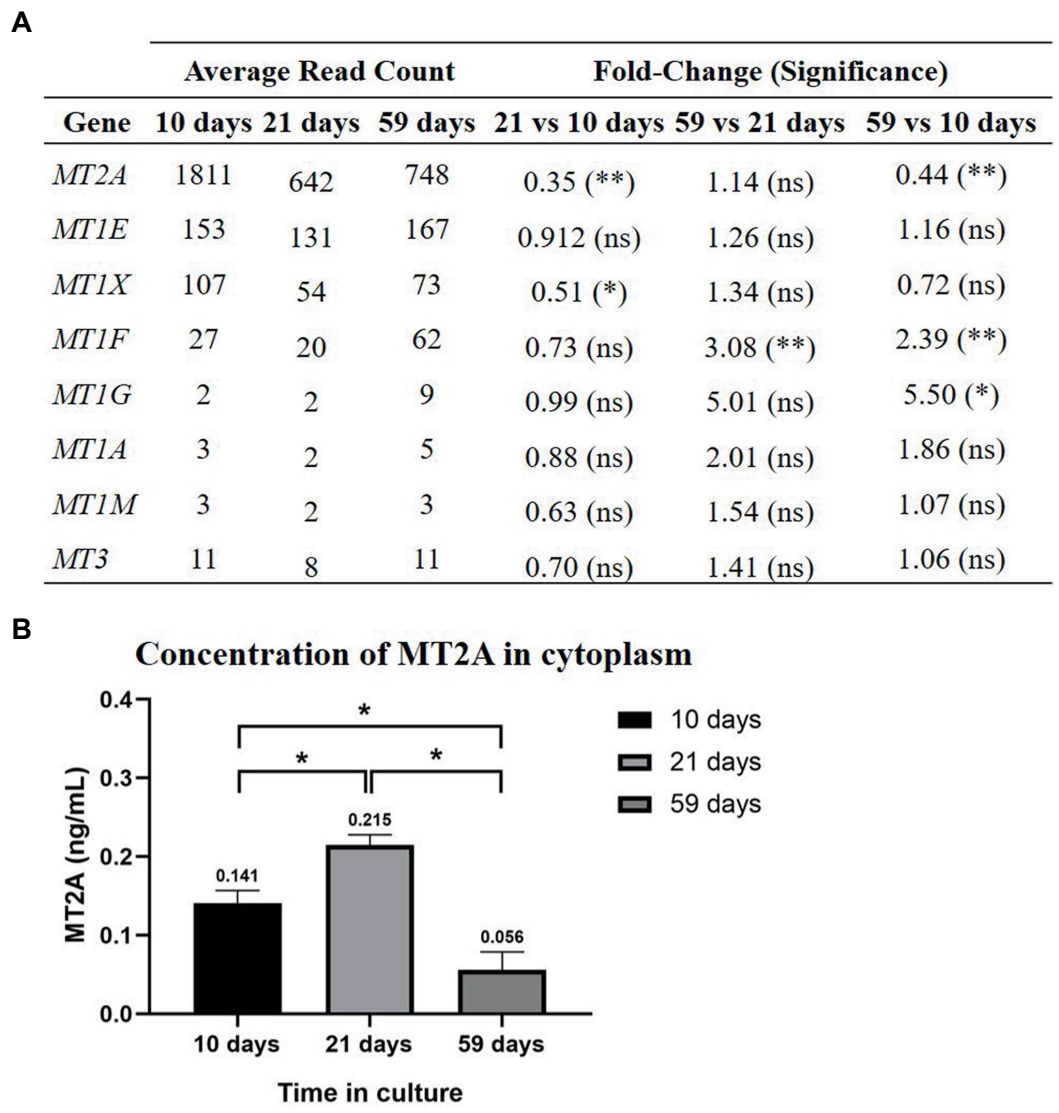
MTs gene expression in the RPE cell cultures at 10, 21, and 59 days **(A)** and MT2A protein concentration in the cytoplasm **(B)**. **(A)** At 59 days there was a downregulation of MT2A and upregulation of MT1F and MT1G. Differential gene expression analysis was carried out following the DESeq2 method (ns: *q*-value >0.05; *: *q*-value <0.05; **: *q*-value <0.01). **(B)** MT2A cytoplasmic concentration increases significantly from 10 to 21 days in culture and decreases from 21 to 59 days in culture. Data is represented as concentration (ng/mL) and error bars depict the standard deviation. *: significant *p*-value of two-way ANOVA (< 0.05).

MT2A, MT1E and MT1X were the most expressed isoforms throughout all times (10, 21, 43, and), although significant changes in gene expression occurred over time (Figure 6A). MT2A was significantly down-regulated, while MT1G and MT1F were up-regulated at the end of the 59 days in culture. The downregulation of MT2A, the most abundant isoform in primary RPE cells, coincides with the decrease in cytoplasmic Zn found at 59 days in culture. MT2A protein levels were determined by ELISA (Figure 6B), which cytoplasmic concentration increases after 21 days (0.215 ± 0.013 ng·mL^−1^) in comparison to 10 days in culture (0.141 ± 0.016 ng·mL^−1^), while from 21 to 59 days (0.056 ± 0.023 ng·mL^−1^) there was a significant decrease in MT2A content, showing same trend as total Zn.

#### 3.2.3. Zinc transporters

Gene expression levels and changes with time of the two families of Zn transporters are shown in Supplementary Table S3 (Supplementary Material): ZnT (ZnT1-10, codified by SCL30 genes), that reduces Zn content in the cytosol by exporting it to the extracellular space or by storing it in organelles; and ZIP (ZIP1-14, codified by SLC39 genes), that promotes Zn import to the cytosol from the extracellular media or storage vesicles. At the end of the 59 days in culture, the coding genes of six ZIP importers had different expression levels than at 10 days in culture, four of them being significantly upregulated (SLC39A12, SLC39A8, SLC39A11 and SLC39A3) and two downregulated (SLC49A10 and SLC39A9). As for the ZnT transporters, six were altered from 59 to 10 days in culture, two of them being significantly upregulated (SLC30A8 and SLC30A10) and four downregulated (SLC30A7, SLC30A6, SLC30A1 and SLC30A9).

### 3.3. Metals monitoring during sub-RPE deposits formation

#### 3.3.1. Copper

**Figure 7:**
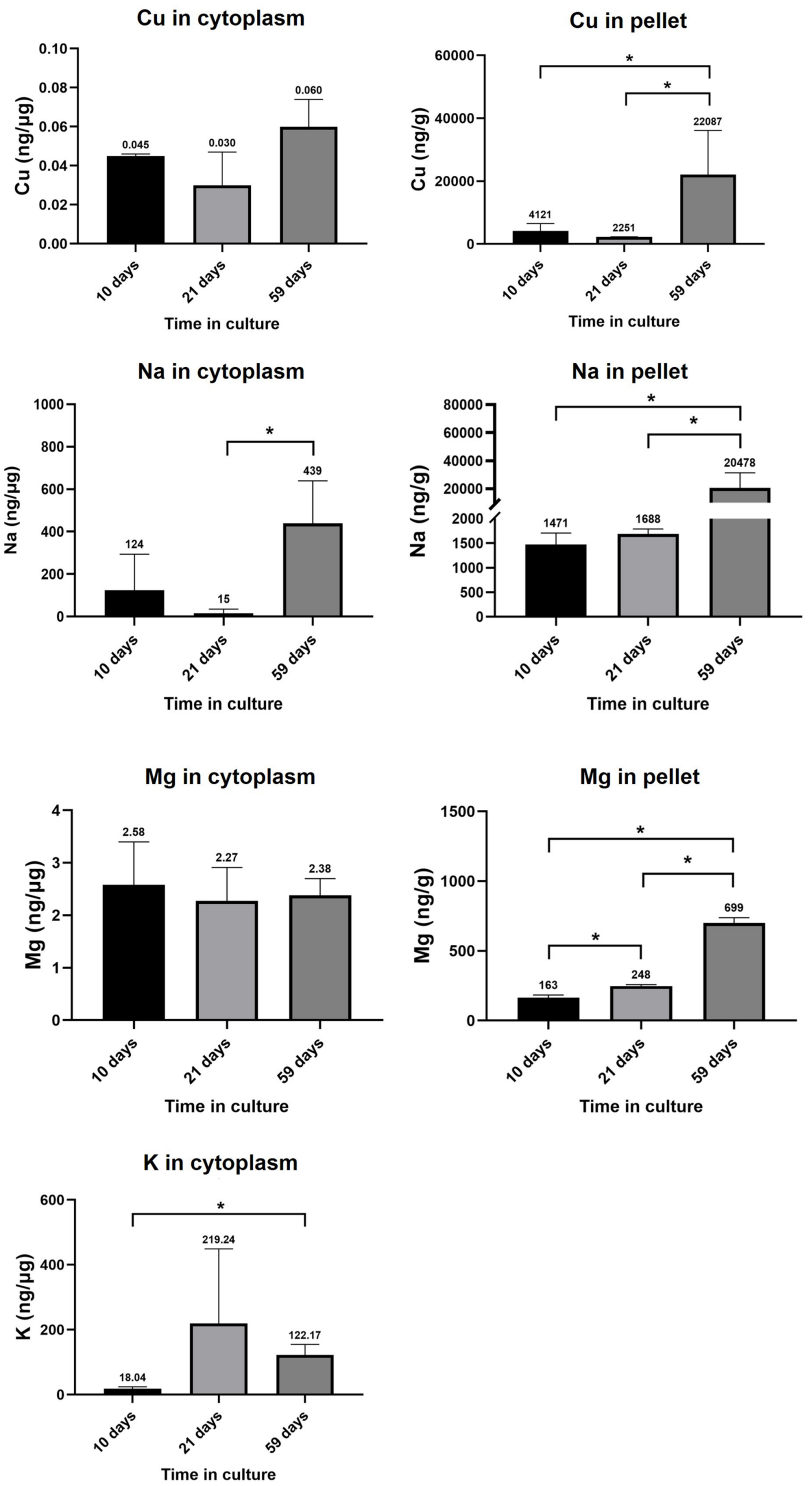
RPE levels of Cu, Na, Mg, and K in cytoplasmic and pellet fractions. Data is represented as concentration (ng·μg^−1^ total protein in the cytoplasm; and ng·g^−1^ sample in the pellet) and error bars depict the standard deviation. ^*^: significant *p*-value of two-way ANOVA (< 0.05).

Cu levels in the cytoplasmic and pellet fractions of RPE cells at 10, 21 and 59 days in culture are depicted in Figure 7. Cu levels in the cytoplasm varied from 0.045 ± 0.001 ng·μg^−1^ (10 days) to 0.030 ± 0.017 ng·μg^−1^ (21 days) and to 0.060 ± 0.014 ng·μg^−1^ (59 days), without statistically significant changes with time. In the pellet fraction, Cu levels varied from 4,121 ± 2,424 ng·g^−1^ (10 days) to 2,251 ± 79 ng·g^−1^ (21 days) and 22,087 ± 14,025 ng·g-^1^ (59 days), observing a significant increase when comparing 59 days in culture with earlier time points (10 and 21 days, value of *p*<0.05).

#### 3.3.2. Sodium

Na levels in the cytoplasm and pellet of cell cultures showed a striking upward tendency with time (Figure 7), increasing 30 times in the cytoplasm of RPE cells between 21 and 59 days in culture (from 15 ± 20 ng·μg^−1^ at 21 days to 439 ± 201 ng·μg^−1^ at 59 days) and 12 times in the pellet (from 1,688 ± 102 ng·g^−1^ at 21 days to 20,478 ± 10,856 ng·g^−1^ at 59 days). The observed increase in intracellular Na could be caused by an altered Na^+^/K^+^-ATPase activity. Expression levels of Na^+^/K^+^-ATPase coding genes in primary RPE cells throughout 10–59 days are depicted in Supplementary Table S4 of Supplementary Material, where most coding genes did not change substantially between 10 and 59 days and only ATP1B2 and ATP1B3 showed a slight up-and down-regulation, respectively.

#### 3.3.3. Magnesium

Mg levels in the cytoplasm and pellet of RPE cells at 10, 21 and 59 days in culture are shown in Figure 7, with no significant changes in Mg concentration in the cytoplasm (varying from 2.58 ± 0.82 ng·μg-^1^ at 10 days to 2.27 ± 0.64 ng·μg^−1^ at 21 days and 2.38 ± 0.32 ng·μg^−1^ at 59 days in culture), whereas Mg content in the pellet significantly increased from 10 days (163.41 ± 19.91 ng·g^−1^) to 21 days (247.57 ± 11.06 ng·g^−1^) and 59 days in culture (699.33 ± 38.60 ng·g^−1^).

#### 3.3.4. Potassium

K levels in the cytoplasm of RPE cells at 10, 21 and 59 days in culture are depicted in Figure 7, observing a significantly increase from 18.04 ± 6.01 ng·μg^−1^ at 10 days to 122.17 ± 32.33 ng·μg^−1^ at 59 days.

### 3.4. Oxidative stress

#### 3.4.1. Expression of antioxidant enzymes

Gene expression analysis of enzymatic antioxidants, including superoxide dismutase (SOD), catalase (CAT), glutathione peroxidase (GPX) and peroxiredoxins (PRDX), indicates the activity of the antioxidant machinery and may indirectly show the cellular protection against oxidative stress. A significant increase in the expression of two isoforms of SOD (SOD2 and 3), CAT, three isoforms of GPX (GPX1, 4 and 8), and PRDX1, had been observed from 10 days to 59 days in culture (see Table 1).

**Table 1:** Temporal changes in the gene expression of antioxidant enzymes in RPE cells at 10, 21, and 59 days in culture.

	Average read count			Fold-change (Significance)		
Gene	10 days	21 days	59 days	21 *vs* 10 days	59 *vs* 21 days	59 *vs* 10 days
*SOD1*	2806	2864	2460	1.09 (ns)	0.85 (ns)	0.93 (ns)
*SOD2*	1740	2378	3252	1.45 (^**^)	1.35 (^**^)	1.97 (^**^)
*SOD3*	583	1067	1720	1.94 (^**^)	1.61 (^**^)	3.13 (^**^)
*CAT*	1015	1249	1377	1.30 (^**^)	1.10 (ns)	1.44 (^**^)
*GPX1*	6419	7397	8796	1.22 (^**^)	1.19 (ns)	1.45 (^**^)
*GPX3*	3492	2588	3471	0.79 (^**^)	1.34 (^**^)	1.05 (ns)
*GPX4*	4633	6605	8432	1.51 (^**^)	1.27 (^*^)	1.93 (^**^)
*GPX7*	967	902	902	0.99 (ns)	0.99 (ns)	0.99 (ns)
*GPX8*	1707	2137	2100	1.32 (^**^)	0.98 (ns)	1.30 (^**^)
*PRDX1*	4427	4953	5424	1.18 (^**^)	1.09 (ns)	1.29 (^**^)
*PRDX2*	6385	6461	6424	1.07 (ns)	1.00 (ns)	1.07 (ns)
*PRDX3*	2047	2198	2189	1.14 (^*^)	0.99 (ns)	1.13 (ns)
*PRDX4*	1958	1867	1758	1.01 (ns)	0.94 (ns)	0.95 (ns)
*PRDX5*	3653	3703	4050	1.07 (ns)	1.09 (ns)	1.18 (ns)
*PRDX6*	4,740	3,323	3,206	0.74 (^**^)	0.96 (ns)	0.71 (^**^)

Fold-Changes and statistical significance test were carried out following the DESeq2 method. ns: q-value >0.05; *: *q*-value <0.05; **: *q*-value <0.01.

#### 3.4.2. Quantification of H_2_O_2_ levels in culture media

Quantification of the reactive oxygen species, i.e., H_2_O_2_, in the apical and basal culture media over time after subtracting no cell control and normalized to total protein, was performed. A significant increase was observed in H_2_O_2_ diffused from the cells to the basal culture media at 59 days in culture when comparing with the basal media at 10 and 21 days (see Supplementary Figure S5 of Supplementary Material).

## 4. Discussion

Retinal Zn homeostasis is affected by ageing and its imbalance can be a risk factor for AMD progression (44, 45). Here we report alterations in Zn levels and MTs, along with increase of oxidative stress and changes in Cu, Na, Mg and K observed in cultured RPE cells producing sub-RPE deposits as result of a physical barrier against regular movement of secreted material. The RPE, an outer retinal single neuroepithelium, constitutes a pigmented monolayer of hexagonal cells that maintain the homeostasis and adhesion of the neurosensory retina (46). The continuous absorption of light, the oxidative milieu and the high metabolic activity of the RPE contributes to its dysfunction, which is exacerbated during ageing. Ageing prompts cellular and molecular changes in all ocular structures, including the RPE, causing varied effects and the onset of neurodegenerative eye diseases (9). The macular region, located near the center of the retina, is particularly susceptible to damage, and the consequent dysfunction of the macular RPE triggers the development of AMD as a result of its degeneration and photoreceptor loss (47). Clinical studies conducted by the Age-Related Eye Disease Study (AREDS) showed that dietary supplementation of Zn and vitamins slows AMD progression, further bringing into attention the possible role of the dyshomeostasis of Zn, and possibly other metals in its pathogenesis (44, 45). Dyshomeostasis of Zn induce various detrimental intracellular events, including oxidative stress, DNA fragmentation, protein misfolding and activation of apoptosis, which leads to neuronal death (48, 49). To study Zn homeostatic alterations and other metals and metalloproteins, we used a human fetal primary RPE cell culture that recapitulates some aspects of early AMD, including progressive accumulation of sub-RPE material (23–25).

The *in vitro* model of AMD has been followed up to 2 months in culture. Cobblestone cell morphology was observed *en face* after 2 weeks, when pigmentation began to be apparent, and was preserved throughout culture maturation [Figure 1; (8)]. Cell–cell and cell-matrix unions have a major role in polarization and barrier function, where tight junctions are the strongest type of unions between cells, creating a fence for protein distribution and paracellular transport (50), indirectly monitored by the TEER follow-up. The TEER has been estimated to be around 206 ± 151 Ω·cm^2^ for the fetal RPE and 79 ± 48 Ω·cm^2^ for the adult RPE *in vivo* (51, 52). In this study, TEER followed an upward tendency on the first month in culture as cell differentiation progressed, reaching a maximum value that was closer to that of *in vivo* RPE on the cell cultures, reaching a pseudo-plateau after 2 months in culture (Supplementary Figure S1 of Supplementary Material). The GO enrichment analysis provided evidence on differences in biological processes during 59 days in culture. RPE cells proliferated (the first week), differentiated, and developed processes normal to RPE, even attempting to communicate with other retinal structures albeit cell cultures singly contained RPE cells (Figure 3).

Proteins participating in the formation of intercellular unions were analyzed at the RNA and protein level (Figure 2). CLDN19 and ZO-1 are essential of the tight junctions and its deficiency has been linked to visual impairment (53). ZO-1 protein was synthesized and recruited early to the cell membrane (at 10 days in culture) before CLDN19, which appeared later (after 21 days in culture). By early binding to junctional sites, ZO-1 facilitates the polymerization of CLDN19, which in turn forms the backbone of tight junctions along with occludins (54). In consonance, the expression of ZO-1 coding gene (TJP1) was higher at 10 days in culture than at later times, which is likely a consequence of the higher requirement for tight junction formation at earlier times. Both ZO-1 and CLDN19 showed an apicolateral localization, revealing that these cells developed a polarized distribution of proteins across their plasma membrane in culture, creating an apicobasal gradient of ions and solutes maintained by the epithelial barrier function. Proteins that are specifically synthesized in the *in vivo* RPE provide an insight into the progress of *in vitro* differentiation of RPE cells (Supplementary Figure S3 of Supplementary Material). Genes of the visual cycle (RGR, LRAT, BEST1, RDH5, RLBP1 and RPE65) and those involved in the biosynthesis of melanin (TYR, DCT, TYRP1 and MITF) were found upregulated, because cells are being differentiated along the first 3 weeks of culture. Interestingly, BEST1, a transmembrane protein participating in Ca homeostasis, neurotransmitter release, and cell volume regulation (55), was preferentially located in the basal area of the cell membrane after 2 months, reflecting an adequate polarization of the RPE cells.

AMD is characterized by the accumulation of molecules in the extracellular space, forming abnormal sub-RPE deposits. In our model system, we looked at several genes related to the capacity of cells to form sub-RPE deposits *in vitro* (23). The cholesterol carrier APOE protein, a known marker of drusen and lipid-rich basal linear deposits (23, 40), was analyzed at gene expression and protein localization levels during RPE cells culture. Punctate APOE deposition has been observed from 3 weeks in culture onwards, with profusion at the end of the culture coincident with the down-regulation of APOE coding gene at 59 days (Figure 3). This alteration may be a regulatory mechanism to avoid an excessive APOE secretion, given that some studies pointed to the existence of compensatory mechanisms in the cell that could replace APOE normal activity (56). It has also been observed specific dysregulation of genes associated with ectopic bone formation after 59 days in culture (OGN, FRZB and THBS4 upregulation and BGN downregulation), although Ca levels were not significantly different along the study (Supplementary Figure S4 of Supplementary Material). These differences may be related with the misregulation of HAP deposition in the membrane of the inserts, although additional analysis are mandatory (40–42). Considering that the cell cultures showed characteristics of sub-RPE deposits accumulation sharing common aspects of early AMD, we studied the homeostatic state of essential elements, including Zn.

Zn is the most abundant trace metal in the retina and is particularly concentrated in the RPE, where it can be bound to proteins, accumulated in secretory vesicles or mitochondria, and free in the cytosol at very low levels (18). In healthy conditions, a robust homeostatic system maintains Zn levels in a nontoxic range. This system is comprised of Zn-regulating proteins like MTs, one of the main components of the Zn muffling molecules capable of binding up to seven atoms of the ion (14, 57, 58), and transporters that shift Zn between the extracellular and intracellular space and between organelles (59). However, during ageing and AMD disease, a decrease in Zn levels and an alteration of its homeostatic system may occur (43). In an *in vitro* context, the temporal changes of Zn concentrations were statistically significant for the decrease of Zn in RPE cells (59 vs. 10 days, 0.2-fold change, Figure 5). Alterations on Zn levels were exclusively observed in the cytoplasmic fraction of RPE, since although Zn decreased in cell nuclei and membranes (0.4-fold, 59 vs. 10 days) these differences were not statistically significant. These data are in agreement with the observed Zn decrease in the RPE with age, suggesting a potential role of altered Zn homeostasis for AMD and confirming the usefulness of this *in vitro* model to evaluate early AMD lesions (19, 23, 43). However, RPE cells were cultured in “Miller medium” containing 1% fetal bovine serum (26), having large Zn buffering capacity. This conventional culture medium renders low Zn availability in the nM range (24), which may implicate a Zn inefficient environment creating a negative gradient for its extracellular release with consequent cytoplasmic decrease. Therefore, Zn homeostatic changes may also be affected by both depositions of extracellular material and culture media with lower Zn bioavailability.

Likewise, the downregulation of the major isoform MT2A at 59 days in culture aligns with the observed decrease in cytosolic Zn (Figure 5). We hypothesize that at early times in the differentiation of RPE (10 days), cells have increased requirements of Zn homeostasis, since Zn levels were the highest in immature cell cultures, and as a result, gene expression of MTs is higher than at later times (59 days), in which RPE cells achieve a stable cell state. Interestingly, the concentration of MTs in the RPE also decreases with age in humans, as occurred in our RPE cell model (14). Lower MTs protein concentration in RPE cells may influence improper Zn homeostasis and contribute to the pathogenic aggregation of proteins and to cell death, since loosely bound Zn and free Zn have been suggested to have signaling functions inside the cell (60). Another relevant aspect was the lack of correlation between the decrease of MT2A gene expression and the increase of MT2A protein abundance at 21 days in culture (comparing to 10 days). This could be the result of the slow regulation of the translation of the MT2A mRNA to protein or irreproducibility (61). Considering that Zn mediates a cross-talk communication between MTs and inflammatory cytokines in a corneal epithelial cell line (14), cytokines gene expression was studied, although both pro-and anti-inflammatory cytokines were indistinctly altered during the RPE cell model differentiation process.

As another component of the Zn homeostasis system, Zn transporters may undergo changes with time that could contribute to the dyshomeostasis of Zn observed during accumulation of sub-RPE deposit material (Supplementary Table S3 of Supplementary Material). Zn influx transporters SLC39A2 and SLC39A4 undergo an age-dependent decrease in human RPE cells (62), but no significant changes were detected in our RPE cell model. Overall, RPE cells after 59 days in culture showed overexpression of four influx and two efflux Zn transporters, and underexpression of two influx and four efflux transporters, which could indicate a stage of Zn sequestration by the cell, although their quantitation is mandatory. The gene SLC39A8, that codes for the transporter ZIP8, is upregulated at 59 days in culture and its overexpression has been associated with the activation of the innate immune response (63), indicating that some inflammation could be taking place in the RPE. The SLC39A12 gene was also significantly highly upregulated after 59 days and codifies the transporter ZIP12, which has been shown to be involved in the cellular response to hypoxia (64).

The observed Zn dyshomeostasis concurred with dysregulation of other metals and metalloproteins. Cu is an essential element for the cellular respiration and activity of several antioxidant enzymes and mainly found in the mitochondria of cells (65), which homeostasis is being partially controlled by MTs (57) and SOD (66). Simultaneous upregulation of SOD (Supplementary Table S3 of Supplementary Material) and Cu (Figure 6; 1.3-fold in the cytoplasm, value of *p*>0.05; 5-fold in the cell nuclei and membrane, value of *p*<0.05) has been observed in RPE cells at 59 days in culture (compared to 10 days). SOD1 and SOD3 isoforms use Cu and Zn as cofactors for superoxide anion dismutation, acting as antioxidant molecule (67). Mice deficient in SOD suffer elevated levels of oxidative species and develop an AMD-like phenotype (68), while knockdown of SOD2 produced pathological lesions similar to those observed in “dry” AMD (69). This upregulation of SOD may contribute to intracellular Cu control and antioxidant defense against the increase of oxidative stress (70). Similarly, the strong increase observed in Na levels, both in the cytoplasm and pellet, may also suggest specific metal dyshomeostasis (Figure 6). Na concentration is tightly regulated to create an ionic gradient across the extracellular and intracellular space of the epithelium that enables the transport of multiple molecules, being involved in osmoregulation, pH buffering, and removal of metabolic products (71). Also, the K levels in the cellular cytoplasm were also increased after 59-days-long culturing, while significant up-regulation of *ATP1B2* and down-regulation of *ATP1B3* were observed (Supplementary Table S4 of Supplementary Material), genes codifying the Na^+^/K^+^-ATPase, the main transporter responsible of the establishment of the Na^+^ gradient, releasing three Na^+^ ions to the extracellular space and introducing two K^+^ ions into the cell (72, 73).

Changes in oxidative stress and antioxidant enzymes have been observed in the RPE cell culture model. Exposure to light, high metabolic activity, and considerable oxygen tension demand for a robust antioxidant system in the eye. This antioxidant system is comprised of several enzymatic and non-enzymatic antioxidants, and it is prone to debilitation with ageing, posing a considerable risk for the development of age-related retinal diseases like AMD (70). Significant upregulation of antioxidant enzymes, including SOD, CAT, GPX and PRDX1 was determined (Table 1), in line with the work of Hsiung et al. (74), observing that polarized RPE expressed higher levels of SOD1 and CAT and higher tolerance to oxidative stress compared with non-polarized RPE. Up-regulation of enzymatic antioxidants underlies the resistance to oxidative stress, as also reported in ARPE19 and hiPSC-RPE cells after 3 weeks in culture as a consequence of its maturation (75, 76). H_2_O_2_ levels in the culture media over time, providing indirect estimation of ensemble reactive oxygen species that can readily diffuse across hydrophobic membranes (70), only showed increased levels in basal media at 59 days. Although the detected increase of oxidative stress could be associated with the observed higher expression of antioxidant enzymes capable of scavenging H_2_O_2_, it should be noted that culture media was renewed each 3 days to replenish nutrients and this may also influence unaltered oxidative stress during cell culture.

In summary, the RPE cell culture model of early AMD showed cobblestone morphology, presence of specific neuroepithelial proteins, higher levels of antioxidant enzymes and deposition of sub-RPE material. The metallo-transcriptomic analysis provided evidences on an altered Zn homeostasis, exacerbated by changes in cytosolic Zn-binding proteins and Zn transporters during the early accumulation of sub-RPE deposits, along with other metals and metalloproteins variations. Further studies with larger sample size, single cell transcriptomics and metallomics approaches and using more complex cell models or carried out in cells derived from AMD postmortem donors will contribute to unravel Zn muffling changes during AMD.

## Data availability statement

The data presented in the study are deposited in the BioStudies repository, accession number S-BSST1057.

## Author contributions

AÁ-B performed the experiments, collected the data, developed the mass spectrometry quantitative methodology for the multi-omics analysis, analyzed the data, and drafted the manuscript. LÁ designed the study, performed RPE cell culturing and follow-up, analyzed the transcriptomic and immunocytochemistry assays and wrote the manuscript. EA assisted with experiments and created Supplementary Figure S2 of Supplementary Material 1. MG performed statistical analysis, offered suggestions, and revised the manuscript. IL analyzed and revised the data, offered suggestions, and edited the manuscript. RP edited the manuscript and coordinated the mass spectrometry analysis. HG-I conceived, coordinated and led the study, interpreted the data and edited the manuscript. All authors have read and agreed to the published version of the manuscript.

## Funding

The Instituto Oftalmológico Fernández-Vega and Fundación de Investigación Oftalmológica acknowledge financial support from the Fundación Rafael del Pino (http://www.frdelpino.es), through the “Cátedra Rafael del Pino.” This work was partially financed through project (PID2019-107838RB-I00/Agencia Estatal de Investigación (AEI)/10.13039/501100011033). AÁ-B acknowledges the FPU grant (Ref. FPU20/00608, Ministry of Universities of Spain).

## Conflict of interest

The authors declare that the research was conducted in the absence of any commercial or financial relationships that could be construed as a potential conflict of interest.

## Publisher’s note

All claims expressed in this article are solely those of the authors and do not necessarily represent those of their affiliated organizations, or those of the publisher, the editors and the reviewers. Any product that may be evaluated in this article, or claim that may be made by its manufacturer, is not guaranteed or endorsed by the publisher.

## Footnotes

^1^ https://github.com/BGI-flexlab/SOAPnuke

^2^ http://www.ccb.jhu.edu/software/hisat

^3^ http://bowtie-bio.sourceforge.net/bowtie2/index.shtml

^4^ http://deweylab.biostat.wisc.edu/rsem/rsem-calculate-expression.html

^5^ http://biosys.bgi.com

^6^ http://www.bioconductor.org/packages/release/bioc/html/DESeq2.html
