# Supplementary material for: Altered zinc homeostasis in a primary cell culture model of the retinal pigment epithelium
Source: Front Nutr. 2023 Apr 17;10:1124987. doi: 10.3389/fnut.2023.1124987 (PMC10149808; doi:10.3389/fnut.2023.1124987)
Supplement: Supplementary file 1 [file Data_Sheet_1.DOCX]

Supplementary Material

**Supplementary Material Description:** The supplementary material contains supplementary data and information of the Material and Methods and Results sections.

# Material and Methods

## Statistical analysis of RNA-seq data

Table S1 collects samples groups in each of the experiments, cell age, sample label and pairwise comparisons.

Table S1. Grouping of samples and pairwise comparison between groups

| Age Group | Sample Name | Pairwise comparisons |
| --- | --- | --- |
| 10-days | 10dA4, 10dB1, 10dB2 | 10-days vs 21-days |
| 21-days | 21dA1a, 21dA2a, 21dA3a | 21-days vs 59-days |
| 59-days | 59dB1, 59dB2, 59dB3 | 10-days vs 59-days |

## Multielemental metallomic analysis by mass spectrometry

Instrumental parameters of ICP-MS are summarized in Table S2.

Table S2. Instrumental parameters used in the FIA-ICP-MS analysis.

| **ICP-MS Agilent 7900** | | |
| --- | --- | --- |
| Plasma parameters | RF Power (W) | 1550 |
|  | Plasma flow (L·min^-1^) | 15 |
|  | Auxiliary gas flow (L·min^-1^) | 0.90 |
| Collision cell parameters | He flow (L·min^-1^) | 0.0045 |
|  | Octopole voltage (V) | -18 |
|  | Quadrupole voltage (V) | -13 |
| Data acquisition parameters | Acquisition mode | Time resolved analysis |
|  | Monitored isotopes | ^23^Na, ^24^Mg, ^31^P, ^44^Ca, ^63,65^Cu, ^64,66,68^Zn, ^69^Ga |
|  | Points per peak | 10 |
|  | Acquisition time per point (s) | 0,31 |
| **ICP-MS Element2** |  |  |
| Plasma parameters | RF Power (W) | 1225 |
|  | Cool gas flow (L·min^-1^) | 15.5 |
|  | Auxiliary gas flow (L·min^-1^)  Sample gas flow (L·min^-1^) | 1.4  1.068 |
| Data acquisition parameters | Acquisition mode | Time resolved analysis |
|  | Monitored isotopes | ^39^K, ^56,57^Fe, ^69^Ga |
|  | Resolution | Medium (R≈4000) |
| **Sample introduction** |  |  |
| Flow injection analysis settings | Injection volume (μL) | 5 |
|  | Replicates | 3 |

# Results

## Barrier function

### Transepithelial electrical resistance

The transepithelial electrical resistance (TEER) has been monitored during the evolution of RPE cell cultures, according to Figure S1.

####

**Figure S1.** Temporal evolution of normalized transepithelial electrical resistance (TEER %) in RPE cell cultures to 100% at day 3. Data points represent the normalized mean TEER values (expressed in %) and the 95% CI, depicted as error bars.

### Intercellular tight junctions

Orthogonal viewing of ZO-1 in 59 days-old RPE cells by confocal microscopy (Figure S2).

**Figure S2.** Orthogonal sections of confocal microscopy images of 59 days-old RPE cells, showing ZO-1 (green) fluorescent signals. Nucleus (blue) were stained with DAPI 0.2 µg·mL^-1^ for 10 minutes at room temperature. Views of orthogonal sections were reconstructed using ImageJ software (National Institutes of Health). ZO-1 is detected in the apical side of the cell membrane, while the nucleus is located basally.

## Expression of specific markers of RPE

List of proteins specifically synthesized in the RPE along days in culture, at both RNA and immunohistochemistry levels (Figure S3).

**Figure S3.** Gene expression of RPE markers (A) and immunolocalization of BEST1 protein (B) in the cell cultures at 10, 21 and 59 days. Differential gene expression analysis was carried out following the DESeq2 method (ns: q-value > 0.05; *: q-value < 0.05; **: q-value < 0.01). **(A)** Several of the analyzed RPE markers are significantly up regulated at the end of the experiment 1 (59 days), with the exception of *PAX6* and *TYR*. **(B)** BEST1 protein was detected in the cell membranes of RPE cells after 21 days, being an increase in signal intensity after 59 days in culture.

## Formation of extracellular deposits

***Calcium levels***

Figure S4 compiles the mean values and standard deviation of Ca levels in cultured RPE cells at 10, 21 and 59 days in culture.

Figure S4. Calcium levels in the cytoplasm and pellet fraction of RPE cells at 10, 21 and 59 days in culture. Data is represented as concentration (ng·μg^-1^ total protein, in the case of the cytosol; and ng·μg^-1^ sample, in the case of the membranes) and error bars depict the standard deviation.

## Zinc homeostatic changes during sub-RPE deposits formation

### Zinc transporters

Gene expression levels and changes with time of ZnT (ZnT1-10) and ZIP transporters (ZIP1-14).

**Table S3.** Temporal changes in the expression of SLC30 and SLC39 Zn transporters in the RPE cells at 10, 21 and 59 days in culture. Fold-Changes and statistical significance test were carried out following the DESeq2 method. ns: q-value > 0.05; *: q-value < 0.05; **: q-value < 0.01.

| **Gene family** | **Gene** | **10 days Average Read Count** | **59 days Average Read Count** | **Fold-Change 59 vs 10 days** |
| --- | --- | --- | --- | --- |
| *SLC39 (influx)* | *SLC39A1* | 3263 | 2828 | 0.92 (ns) |
|  | *SLC39A3* | 1015 | 1163 | 1.21 (*) |
|  | *SLC39A4* | 151 | 109 | 0.76 (ns) |
|  | *SLC39A5* | 1 | 1 | 0.72 (ns) |
|  | *SLC39A6* | 2513 | 2519 | 1.06 (ns) |
|  | *SLC39A7* | 4028 | 4096 | 1.08 (ns) |
|  | *SLC39A8* | 322 | 407 | 1.33 (**) |
|  | *SLC39A9* | 1506 | 1166 | 0.82 (*) |
|  | *SLC39A10* | 933 | 465 | 0.52 (**) |
|  | *SLC39A11* | 193 | 242 | 1.32 (*) |
|  | *SLC39A12* | 228 | 2606 | 12.06 (**) |
|  | *SLC39A13* | 3415 | 3518 | 1.09 (ns) |
|  | *SLC39A14* | 1307 | 1332 | 1.08 (ns) |
| *SLC30 (efflux)* | *SLC30A1* | 526 | 353 | 0.71 (**) |
|  | *SLC30A2* | 8 | 13 | 1.78 (ns) |
|  | *SLC30A3* | 4 | 1 | 0.27 (ns) |
|  | *SLC30A4* | 329 | 306 | 0.98 (ns) |
|  | *SLC30A5* | 947 | 853 | 0.95 (ns) |
|  | *SLC30A6* | 739 | 437 | 0.62 (**) |
|  | *SLC30A7* | 915 | 512 | 0.59 (**) |
|  | *SLC30A8* | 57 | 358 | 6.67 (**) |
|  | *SLC30A9* | 1612 | 1166 | 0.82 (*) |
|  | *SLC30A10* | 666 | 1538 | 2.43 (**) |

### Metals monitoring during sub-RPE deposits formation

#### Sodium homeostasis: Na^+^/K^+^-ATPase

Expression of Na^+^/K^+^-ATPase coding genes in primary RPE cells throughout 10-59 days are shown in Table S4.

**Table S4.** Expression of Na^+^/K^+^-ATPase genes at 10, 21 and 59 days in culture. Fold-Changes and statistical significance test were carried out following the DESeq2 method. ns: q-value > 0.05; *: q-value < 0.05; **: q-value < 0.01

|  | **Average Read Count** | | | **Fold-Change (Significance)** | | |
| --- | --- | --- | --- | --- | --- | --- |
| **Gene** | **10 days** | **21 days** | **59 days** | **21 vs 10 days** | **59 vs 21 days** | **59 vs 10 days** |
| *ATP1B1* | 5279 | 6502 | 5693 | 1.31 (**) | 0.87 (ns) | 1.14 (ns) |
| *ATP1B2* | 281 | 699 | 945 | 2.63 (**) | 1.34 (*) | 3.55 (**) |
| *ATP1B3* | 1566 | 1175 | 1037 | 0.80 (**) | 0.88 (ns) | 0.70 (**) |
| *ATP1B4* | 21 | 30 | 17 | 1.48 (ns) | 0.56 (ns) | 0.83 (ns) |
| *ATP1A1* | 10874 | 10074 | 9555 | 0.98 (ns) | 0.94 (ns) | 0.93 (ns) |
| *ATP1A2* | 14 | 4 | 5 | 0.34 (ns) | 1.22 (ns) | 0.41 (ns) |
| *ATP1A3* | 2251 | 4090 | 2504 | 1.92 (**) | 0.61 (**) | 1.18 (ns) |

## Oxidative stress

### Quantification of H_2_O_2_ levels in culture media

Quantitative analysis of the reactive oxygen species, i.e., H_2_O_2_, in the apical and basal culture media over time is shown in Figure S5.

**Figure S5.** H_2_O_2_ levels in the apical and basal culture media of cell cultures at 10, 21 and 59 days. Y-axis represents the percentage of luminescence of H2O2-induced luciferase reaction with respect to the blank (fresh cell culture) and normalized tot total protein. Data are depicted as mean column bars and SD error bars. According to the two-way ANOVA test, significant differences are indicated by asterisks (*).

**
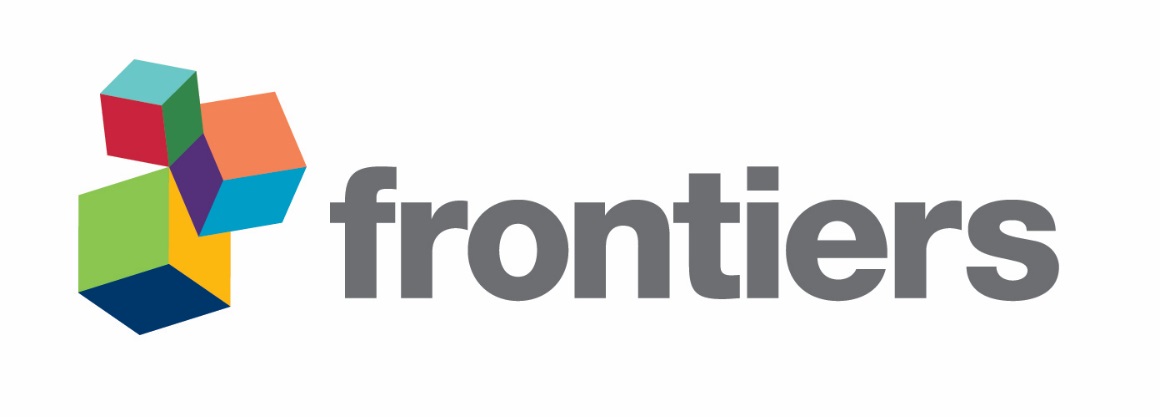
**
